# Epigenetics and ultraviolet radiation: Implications for skin ageing and carcinogenesis

**DOI:** 10.1002/ski2.410

**Published:** 2024-07-05

**Authors:** Bethany M. Barnes, Anya Shyne, David A. Gunn, Christopher E. M. Griffiths, Rachel E. B. Watson

**Affiliations:** ^1^ Centre for Dermatology Research Faculty of Biology, Medicine and Health Manchester Academic Health Science Centre The University of Manchester Manchester UK; ^2^ Unilever R&D at King's College London Guy's Hospital Great Maze Pond London UK; ^3^ A*STAR Skin Research Laboratory (A*SRL) Agency for Science Technology and Research (A*STAR) Singapore Singapore

## Abstract

Recent published data have highlighted the importance of epigenetics in the response of the skin to recreational and therapeutic ultraviolet radiation (UVR) exposure. ‘Epi’—from the Greek επί, meaning over, outside of or around—relates to the chemical modifications that occur on top of the DNA sequence (for example, DNA methylation) and its associated proteins (e.g. histone modifications, including methylation, acetylation and phosphorylation). These epigenetic processes, collectively called the ‘epigenome’, dictate the three‐dimensional conformation of the DNA, thus impacting upon gene expression and genomic stability. Given that epigenetic changes are long‐lived and mitotically heritable, an accumulation of epigenetic perturbations likely influence the pathogenesis of the chronic consequences of UVR exposure, including photoageing and skin cancer risk. In this review, we describe the multifarious epigenetic effects elicited by UVR in the skin. We further speculate on the underlying molecular mechanisms that may direct epigenetic changes, such as oxidative stress and changes in metabolism, and their impact on skin health and disease.

## Graphical abstract

The accumulation of ultraviolet radiation (UVR)‐induced epigenetic changes likely influences the chronic consequences of UVR exposure. In this review, we describe the perturbations in DNA methylation and histone modifications that are observed following short‐term and long‐term UVR exposure, in both healthy and malignant skin. In addition, we speculate on the underlying biological mechanisms that may direct these changes, as well as how these changes may contribute to skin ageing and carcinogenesis.

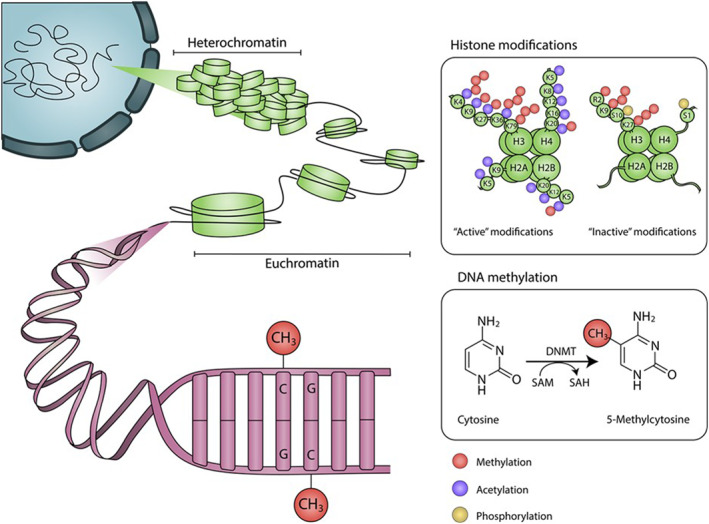

**What is already known?**

- Epigenetic mechanisms, such as histone modifications, DNA methylation, and RNA methylation, have an important role in genomic regulation and may be influenced by environmental factors, including ultraviolet radiation (UVR).

**What does this study add?**

- There is a divergence in the epigenetic landscape between photoaged skin, caused by long‐term exposure to UVR, and skin that has naturally aged with minimal exposure to harmful environmental factors.
- The global loss of DNA methylation observed in photoexposed skin may be due to decreased folate levels leading to a lack of methylation deposition by the methyl donor, SAM. Additionally, oxidative damage to DNA may result in poor recognition of DNA methylation by the maintenance DNA methyltransferase, DNMT1.
- Methylated cytosines are an important component in UVR‐induced mutagenesis and the subsequent development of cutaneous malignancies due to the preferential formation of cyclobutane pyrimidine dimers at these sites.
- Different epigenetic features are present in melanoma, cutaneous squamous cell carcinoma and basal cell carcinoma. However, hypermethylation of CpG islands associated with tumour suppressor genes is common across each of these skin malignancies. Additionally, dysregulation of the histone regulator, EZH2, has been implicated in all.

## 1 BACKGROUND

The skin forms the first line of defence against a daily onslaught of environmental stressors, protecting against physical, chemical and radiation insult. Its ability to produce an effective barrier is dependent upon the architecture of its two main layers: the epidermis, a highly self‐renewing epithelium that forms the outer layer of the skin and the underlying dermis, which consists of a rich supportive extracellular matrix (ECM). Ultraviolet radiation (UVR), present in natural sunlight and artificial sources, is associated with injurious effects that occur both in the acute (erythema, commonly called “sunburn”) and chronic phases (for example, photoageing and skin carcinogenesis) of UVR exposure. Excessive UVR exposure is arguably the greatest driver of premature skin ageing in lightly pigmented individuals and has been significantly correlated with facial skin wrinkling and wrinkle depth.^1^, ^2^ Furthermore, UVR is a complete carcinogen defined by its ability to both initiate and promote cancer. In an ageing population, research into the biological mechanisms behind UVR‐induced skin ageing is essential to aid and reduce the burden of its associated comorbidities, such as skin malignancies, autoimmune diseases, fungal infections and pruritus,^3^ along with the emotional burden associated with the deterioration of skin appearance.^4^

While overexposure of the skin to UVR is of detriment to human health, small levels of exposure produce beneficial effects. The major source of vitamin D in humans is exposure of the skin to ultraviolet B (UVB) radiation.^5^ Furthermore, phototherapeutic regimens are an effective treatment modality for the management of inflammatory dermatoses.^6^, ^7^ This therapy is thought to act via immune suppression, alteration in cytokine expression and cell cycle arrest, which suppresses disease progression and shows clear changes in the epigenome that reflect its effect.^8^

Emerging evidence advocates a role for the epigenome in the skin's response to UVR.^9^, ^10^, ^11^, ^12^, ^13^, ^14^ The archetypical examples of epigenetic mechanisms are the post‐translational modification of histones and DNA methylation (DNAm). By tempering chromatin higher‐order structure, these processes collaborate in the regulation of the associated genetic material without changes to the DNA sequence. Epigenetic changes tend to be long‐lived and mitotically heritable; therefore, it is likely that perturbations associated with chronic UVR exposure may drive molecular changes that result in photoaged skin and increased skin cancer risk. Herein, we review recent studies across skin ageing and carcinogenesis that have revealed perturbations at multiple levels of the epigenetic landscape. We also speculate on the underlying cellular and molecular mechanisms that may direct these epigenetic changes, such as oxidative stress and changes in metabolism, which are known to occur following UVR exposure.

## 2 OVERVIEW OF EPIGENETIC PROCESSES

Conrad Waddington coined the term ‘epigenetics’ in 1942 in a founding paper of the field,^15^ in which it was proposed to describe the interactions between the genome and the environment that interrelate to produce an observed phenotype. Its definition has evolved since its early usage, now defined by the stable inheritance of alternative chromatin states that occur in the absence of changes in the underlying DNA sequence. Epigenetic dynamics are important for numerous biological phenomena, including cell‐type specific gene expression and higher order organization of chromatin, as well as more specific roles such as X‐chromosome inactivation,^16^, ^17^ genomic imprinting^18^, ^19^ and suppression of retroelement transposition.^20^

The epigenome is believed to be the interface between the genome and the environment to which an individual is exposed. Localised and genome‐wide signatory changes in the epigenome have been identified for several lifestyle factors, including chronic UVR exposure,^9^, ^10^, ^11^, ^12^, ^13^, ^14^ tobacco smoking,^21^, ^22^, ^23^, ^24^ alcohol consumption^25^, ^26^, ^27^ and obesity.^28^ This indicates that lifestyle factors are important in the process of healthy ageing. Correspondingly, the degree of epigenetic change between monozygotic twin pairs has been shown to increase in pairs living more separate lives, despite being epigenetically indistinguishable in early infancy.^29^, ^30^ Furthermore, age predicted by the DNA methylome has been found to be significantly influenced by the environment, with relatives that live together showing a highly correlated DNA methylation age which diverges slowly with time lived apart.^31^

### 2.1 Histone modifications

Chromatin describes the structure of DNA and proteins, consisting of repeating units called nucleosomes, which allow the DNA to be packaged concisely into the cell's nucleus. Within a nucleosome, DNA is wrapped around an octameric cylindrical structure consisting of two copies of each of the four core histone proteins (H2A, H2B, H3 and H4).^32^ Stretches of DNA connect the nucleosomes, bound at the entry and exit site of the core histone particle by the linker histone (H1), which facilitates inter‐nucleosomal interactions.^33^ Long N‐terminal tail domains of histones protrude from the nucleosome structure, making them available for chemical modification via acetylation, methylation, phosphorylation, sumoylation, ubiquitylation, deamination, proline isomerisation and adenosine diphosphate ribosylation.^34^ Together, these modifications form the ‘histone code’, which is deciphered by proteins that bind to specific modifications and subsequently regulate transcription via chromatin remodelling and the condensation of nucleosomes; this regulation has been extensively reviewed.^34^, ^35^ Consequently, chromatin is arranged into open genomic regions that are transcriptionally active (euchromatin) and tightly packed regions of suppressed gene expression (heterochromatin).^36^, ^37^, ^38^, ^39^

**FIGURE 1:**
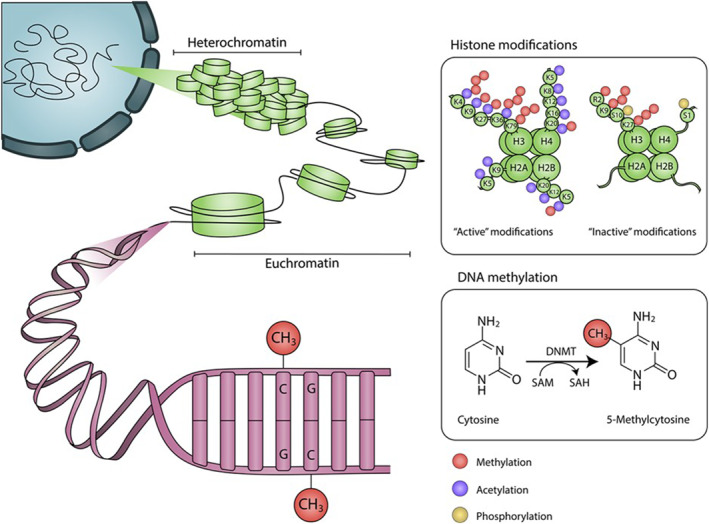
Schematic of epigenetic modifications. The nucleosome is the repeating unit of chromatin, an octameric cylindrical structure consisting of two copies of each of the four core histone proteins (H2A, H2B, H3 and H4; shown in green) that winds ∼147 bp of DNA in 1.7 superhelical turns. Heterochromatin is a form of chromatin that is densely packed—as opposed to euchromatin, which is loosely packaged. Epigenetic modifications: Histone modifications predominantly occur on the N‐terminal tails, consisting of methylation (red), acetylation (blue) and phosphorylation (yellow), among other modifications. Traditionally associated ‘active’ and ‘inactive’ modifications are indicated. DNA methylation occurs predominantly in the cytosine‐phosphate‐guanine (CpG) context, catalysed by a family of DNA methyltransferases (DNMTs) that utilise S‐adenosyl methionine (SAM) as a methyl donor, and catalyse its formation to S‐adenosylhomocysteine (SAH).

This differential regulation of the chromatin can be exemplified by expanding on the specific chemical and molecular mechanisms underlying histone acetylation and methylation. Histone acetylation is controlled by enzymes that catalyse the addition of an acetyl group to the ε‐amino group of a lysine side chain (histone acetyl transferases [HATs]) and those that remove it (histone deacetylases [HDACs]). Lysine residues possess a positive charge, which is neutralised upon addition of an acetyl group. This neutralisation may prevent tight folding of nucleosomal arrays and increase accessibility of the DNA, as demonstrated by an increased sensitivity to DNase I.^40^ Accordingly, broad histone acetylation is known to mark domains permissive to gene transcription (Figure 1).

Histone methylation occurs on the three basic residues: lysine,^41^ arginine^42^ and, less frequently, histidine.^43^, ^44^ Enzymes that catalyse the addition of a methyl group are the histone methyltransferases, divided into three families: the SET (Su(var)3‐9, Enhancer of Zeste, Trithorax)‐domain‐containing proteins, DOT1 (disruptor of telomeric silencing‐1)‐like proteins and protein arginine N‐methyltransferases (PRMTs). The SET‐domain‐containing proteins and DOT1‐like proteins catalyse the transfer of a methyl group from S‐adenosyl‐L‐methionine (SAM) to a lysine's ε‐amino group; PRMTs catalyse the transfer of a methyl group from SAM to the ω‐guanidino group of arginine. While acetylation occurs only singly, lysine may be modified by mono‐, di‐ or tri‐methylation. Similarly, arginine may be modified by mono‐ and symmetric or asymmetric di‐methylation. Histone methylation can be a marker of both active and inactive regions of chromatin (Figure 1). For example, methylation of histone 3 at lysine 9 (H3K9me) occurs widely throughout the inactivated female X chromosome^45^; methylation of lysine 4 (H3K4me) on the same histone, however, is a marker for actively transcribed genes.^46^

### 2.2 DNA methylation

DNA methylation occurs almost exclusively in the palindromic Cytosine‐phosphate‐Guanine (CpG) dinucleotide context, whereby a methyl group from SAM is covalently attached at the fifth carbon position of cytosine, forming 5‐methylcytosine (5mC) (Figure 1). This is catalysed by a family of DNA methyltransferases (DNMTs). De novo DNAm is established by the activity of the methyltransferases DNMT3A and DNMT3B, whereas DNMT1 displays a preference for hemimethylated substrates, and hence is the proposed maintenance methyltransferase which localises to replication foci during the S phase of the cell cycle.^47^ Loss of methylation at CpG sites occurs by hydroxylation of 5mC to 5‐hydroxymethylcytosine (5hmC), catalysed by the ten‐11 translocation (TET) family of proteins.^48^, ^49^ Iterative oxidation of 5hmC generates 5‐formylcytosine and 5‐carboxylcytosine, which are excised by thymine DNA glycosylase: this engages the base excision repair pathway to restore an unmodified cytosine.^49^

Approximately 70% of gene promoters contain CpG islands (CGIs), representing regions of approximately 1000 bp with an elevated C and G content.^50^ Methylation in this genomic context is typically associated with gene silencing; hence, the majority of CGIs are hypomethylated to allow for an open chromatin structure that facilitates gene expression.^51^ In non‐CGI genomic loci, high levels of methylation are also associated with repression of retroviral elements,^52^ enhancer activity^53^ and isoform expression.^54^ However, to say that DNAm is a repressive epigenetic mark would be an oversimplification: it has also been associated with gene activation when situated within gene bodies^55^ and some gene promoters, where certain transcription factors show an affinity towards methylated regions.^56^ In addition, intermediate levels of DNAm at distal enhancers have been shown to correlate with a primed or activated enhancer state.^53^ Furthermore, genes with hypomethylated CGIs may still be transcriptionally repressed.^57^, ^58^

Methylation of adenine, forming N6‐methyladenine, has more recently been demonstrated in the human genome. It is enriched in the mitochondrial genome and exon regions of nuclear DNA, where its density is positively associated with gene transcription.^59^ This modification has not extensively been investigated in the human genome and currently little data is available for the skin.

### 2.3 RNA methylation

Methylation of RNA also occurs, with by far the most common epitranscriptomic mark being the methylation of adenosine (N6‐methyladenosine) in human cells.^60^ This RNA modification is associated with RNA translational efficiency in the skin, particularly in genes and pathways linked to basal cell carcinoma (BCC), including Wnt signalling.^61^

Ribonucleic acid methylation is carried out by a methyltransferase complex that includes methyltransferase‐like (METTL) proteins 3/14, Wilms tumour 1 associated protein and co‐factors.^62^ The expression of METTL3 is higher in squamous cell carcinoma (SCC) and its ablation impairs the cells' ability to form tumours.^63^ In addition, METTL3 expression is also increased in melanoma cells, resulting in an accumulation of matrix metalloproteinase (MMP) 2 and N‐cadherin which enhances the invasive ability of tumour cells.^64^ Furthermore, N6‐methyladenosine at the 5′ end of mRNAs associates with upregulated genes in drug‐resistant melanoma cells, highlighting its varied effect on translation depending on the position of the N6‐methyladenosine within the mRNA. Hence, the altered regulation of RNA methylation likely enables dysregulation of cell behaviour in skin cancers.

## 3 THE SUN‐EXPOSED EPIGENOME IN HEALTHY SKIN

The skin ages as a result of endogenous factors (i.e. hormonal, genetic and metabolic factors), such as free radical damage from cell respiration, in a process known as intrinsic ageing. This is generally characterised by pale, finely wrinkled skin, associated with dryness (xerosis), itch (pruritus), increased susceptibility to infections, autoimmune disease, and vascular complications.^65^ This deterioration of cutaneous function is exacerbated by exposure to exogenous, environmental factors, such as UVR from sunlight, smoking, pollution and diet; together, this is known as extrinsic ageing. Likely the most common and damaging of exogenous factors to cutaneous function is UVR, which results in dyspigmented, deeply wrinkled and thickened skin with an increased risk of cutaneous malignancy in lightly pigmented individuals.^65^, ^66^, ^67^ This is termed photoageing.

### 3.1 Differential DNA methylation occurs with acute UVR exposure and in photoageing

Photoageing is often described as an acceleration of the natural intrinsic ageing process. However, recent studies have uncovered a divergence in the DNAm patterns observed in chronically photoexposed skin in comparison to photoprotected intrinsically aged skin suggesting that the ageing process deviates following chronic sun exposure. In intrinsic skin ageing, studies show that there is an overall general trend towards hypermethylation across the genome.^9^, ^10^, ^13^, ^68^ In the skin that has been chronically photoexposed, two early studies suggested that very minimal changes were observed in the methylome of photoexposed skin compared to photoprotected.^13^, ^69^ The first of these examined the difference between skin biopsies taken from the photoexposed outer forearm and the photoprotected inner forearm using the early generation Illumina Infinium HumanMethylation27 BeadChip array.^13^ The ∼27,000 probes of this array mainly target methylation sites within CGI regions of the genome.^70^ Similarly, the second of these studies investigated the effects of UVB radiation on human keratinocytes using a CGI‐focused methylation CpG island recovery assay.^69^ However, when using approaches that instead targeted non‐CGI areas of the genome, such as the Illumina Infinium HumanMethylation450 BeadChip array, it was found that expansive regions of hypomethylation, primarily outside CGIs and within known heterochromatic regions associated with cancer, were a feature of chronically photoexposed epidermal biopsies compared to photoprotected.^9^, ^71^ Furthermore, hypomethylation correlated with the degree of photodamage, as measured by Griffiths' photonumeric scale,^9^ highlighting a link with photo‐driven physiological changes in the skin. This suggests that hypomethylation largely outside CGIs is the main characteristic of UVR exposure in vivo (Table 1). Therefore, future work should address whether DNAm changes occur in isolated cell systems in heterochromatic regions of the genome, such as repetitive elements and transposons, that were not interrogated previously. In addition, such in vitro studies could be utilised to determine whether such changes are due to direct UVR effects on DNA and chromatin or due to secondary mechanisms such as damage to nuclear lamina proteins, inflammatory processes or photodegradation of metabolites.

**TABLE 1:** A summary of the epigenetic modifications associated with photoageing, melanoma, cutaneous squamous cell carcinoma, and basal cell carcinoma.

	Summary of epigenetic modifications
Photoageing, healthy skin	
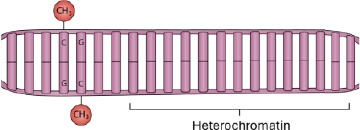	Hypomethylation of non‐CpG island, heterochromatic regions.
	Opposed to slight trend in hypermethylation observed in intrinsic ageing.
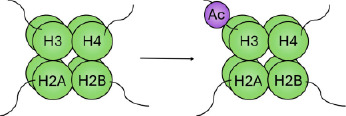	Global increase in transcriptionally active histone acetylation following acute UV exposure, chronic photoexposure, and in intrinsic ageing.
Melanoma	
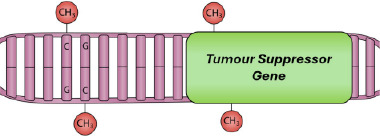	Hypermethylation at the promoter of tumour suppressor genes.
	Global hypomethylation in heterochromatic regions.
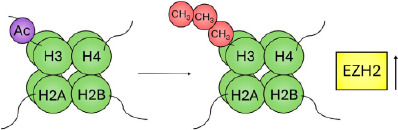	Range of aberrant histone modifications, including transcriptionally repressive histone hypoacetylation and H3K27me3 and H3K9me3 at tumour suppressor genes and Hox genes. Epigenetic regulator, EZH2, upregulated.
Cutaneous squamous cell carcinoma	
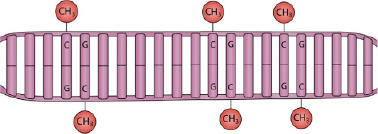	Hypermethylation at the promoter of tumour suppressor genes.
	Global loss of methylation in early SCC formation, followed by global hypermethylation from low‐risk to high‐risk tumour.
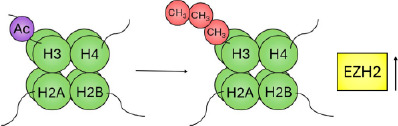	Range of aberrant histone modifications including differential H3K27ac and H3K27me3, which were linked to immune dysregulation. Several regulators of histone modifications linked to SCC, for example, EZH2 and KMT2D.
Basal cell carcinoma	
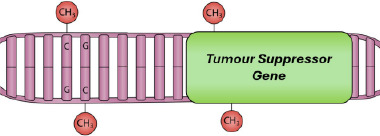	Hypermethylation at the promoter of tumour suppressor genes.
	Little information available with epigenetics of BCC not extensively studied.
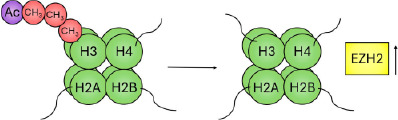	Differential H3K27ac linked to abnormal epidermal homoeostasis.
	Upregulation of EZH2 linked to aggressive BCC, whilst increased H3K27me3 linked to mild BCC.

*Note*: Both DNA methylation and histone modifications are described. Please note that this summary relates to the main points discussed in this article and is not an exhaustive list of the epigenetic modifications that occur for each condition.

With respect to the effect of UVR on the dermal methylome, previous studies have found that there are less distinct changes in methylation patterns in the chronically sun‐exposed dermis in comparison to the epidermis.^9^, ^13^ More recently, it has been observed that the acute DNAm response to UVR in the dermis differs to the chronic response.^12^ It was found that at 2‐day post‐exposure to ultraviolet A (UVA), fibroblasts showed predominantly hypomethylation in active gene regions, some of which were associated with age‐related genes; another study observed similar hypomethylation within gene bodies (30 min and 3 h post‐UVR exposure) of UVR‐exposed fibroblasts, which led to the upregulation of DNA damage response genes.^72^ After a 7‐day recovery period, the majority of these sites had returned to their initial state and, instead, hypermethylation in inactive gene regions was predominantly observed and associated with some developmental genes.^12^ These alterations persisted, suggesting that a more chronic response to UVR in the dermis is observed as hypermethylation, the opposite of that seen in the photoaged epidermis. These results also indicate that studies that focus only on the immediate effects of UVR to the methylome cannot verify whether such acute exposures recapitulate chronic in vivo exposures.

**FIGURE 2:**
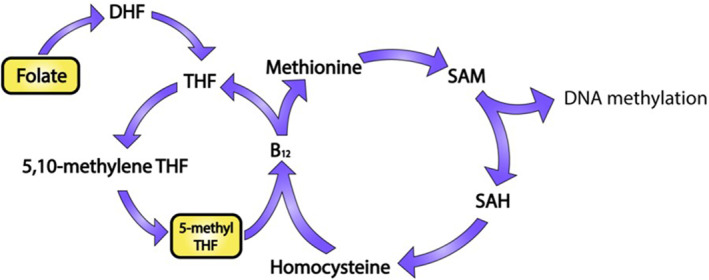
Folate‐dependent one‐carbon metabolism and its photosensitive intermediates. DNA methyltransferases utilise S‐adenosyl methionine (SAM) as a methyl donor, forming S‐adenosylhomocysteine (SAH). S‐adenosylhomocysteine is enzymatically converted to homocysteine, which can be remethylated, forming methionine, using a methyl group from 5‐methyltetrahydrofolate (5‐methyl THF) and utilising vitamin B12 as cofactor. Experimental and in vivo evidence suggests folate and 5‐methyl THF are sensitive to photodegradation (see text for details).

The biological mechanisms behind these epigenetic changes are not well understood. It has been suggested that the depletion of the global methyl donor, SAM, is one route in which the global hypomethylation observed following photoexposure of the epidermis occurs. Folate and its reduced forms are essential intermediaries which, in the form of 5‐methyltetrahydrofolate, participate in one‐carbon metabolism reactions that replenish the methyl donor, SAM (Figure 2). Early in vitro data have demonstrated that human plasma exposed to UVA radiation displays a statistically significant reduction in folate concentration.^73^ In addition, 5‐methyltetrahydrofolate, the main circulatory form of folate, is degraded by UVB^74^ and is sensitive to oxidation by UV‐derived reactive oxygen species (ROS).^75^ Fair‐skinned individuals undergoing photochemotherapy displayed low serum folate concentrations, giving early evidence of folate depletion occurring in vivo.^73^ Another study found that serum folate is sensitive to seasonal fluctuations of solar UVR: significantly lower levels of serum folate are observed in the summer months when UVR levels are at their highest.^76^ Furthermore, the same study found individuals are at a higher risk of folate deficiency in summer. Whilst serum folate reflects the short‐term effect of UVR on folate levels, red blood cell folate indicates the long‐term effect, and this was also found to decrease in response to 4 months of high environmental UVR in elderly subjects in Australia.^77^ The epidermis is an avascular structure, receiving nourishment from vessels in the underlying dermis. The inefficient delivery of micronutrients such as folate, and its potential degradation upon UVR exposure, suggests that serum folate deficiencies would be equal, if not exacerbated, in the epidermis of the skin. While there is currently a dearth of information regarding one‐carbon metabolism reactions in the skin, determining the degree of folate depletion in the skin cells may, in part, explain the propensity of photoexposed skin to be globally depleted of DNAm.

**FIGURE 3:**
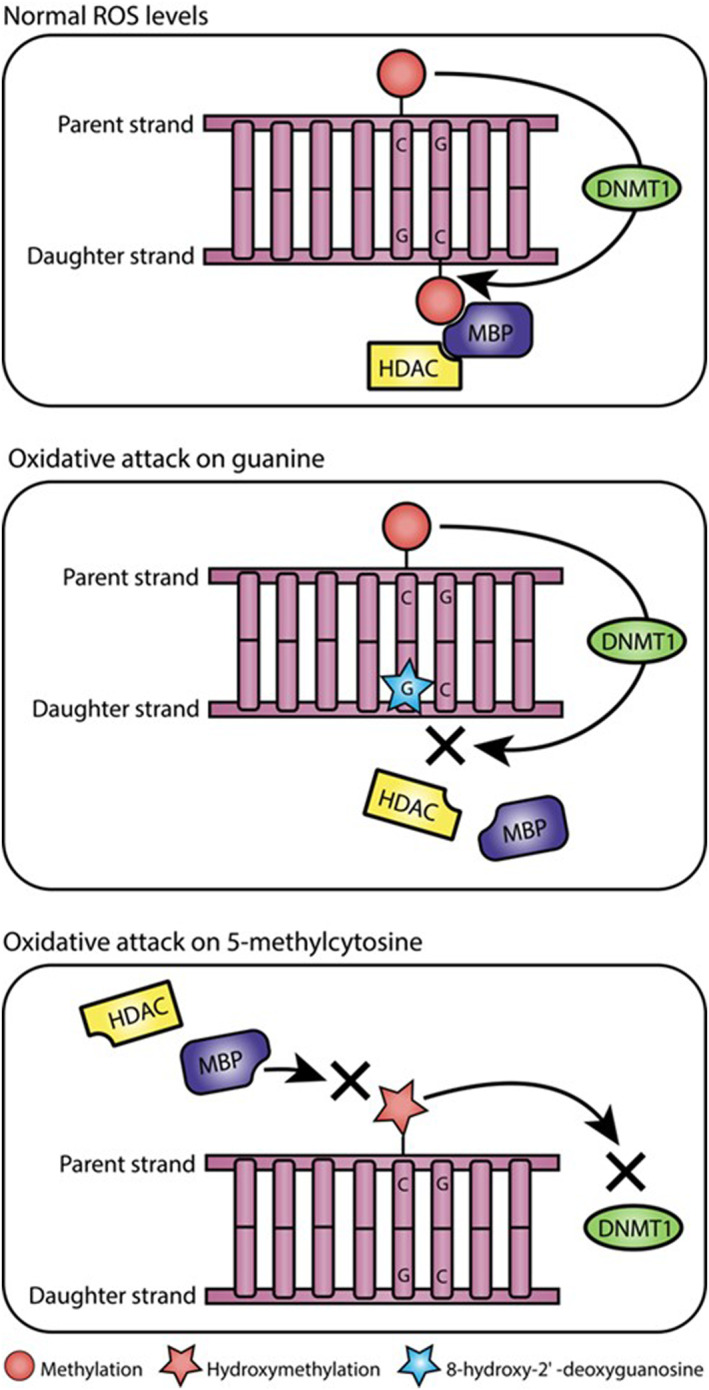
Oxidative DNA damage and epigenetic modifications. Normal ROS levels: DNA methyltransferase 1 (DNMT1) recognises sites of methylation on the parent strand of DNA and catalyses the transfer of a methyl group to cytosine residues on the daughter strand of DNA during DNA replication. Oxidative DNA damage occurring within a CpG dinucleotide may negatively affect recognition of DNA methylation by DNMT1 of the parental DNA strand and/or inhibit DNMT1s ability to deposit methylation on the daughter strand. This can be seen in the two following panels of the figure: Oxidative attack on guanine‐ DNA methylation mediates chromatin organization via binding of MBPs that recruit histone modifying enzymes. Incorporation of 8‐OHdG and 5hmC in the MBP recognition sequence significantly inhibits the binding of MBP, meaning loss of DNA methylation may dysregulate multiple layers of epigenetic control. Oxidative attack on 5‐methylcytosine‐ Oxidative damage affecting methylated cytosine forms the oxidative by‐product 5hmC; since 5hmC is recognized poorly by DNMTs, DNA methylation may be lost at 5hmC sites.

Another mechanism in which DNAm levels may change in response to UVR is via oxidative attack on CpG sequences. One mechanism through which UVR elicits its damaging effects is through the depletion of cellular antioxidants and generation of ROS, including hydrogen peroxide, hydroxyl radical, singlet oxygen and peroxyl radicals. Oxidative stress occurs when ROS exceed the capacity of protective antioxidant systems, leading to oxidative attack on cellular structures, including DNA.^78^ The formation of DNA base adducts within a CpG dinucleotide sequence has been shown to negatively affect DNMTs ability to recognise the hemi‐methylated DNA template during semi‐conservative DNA replication, and/or inhibit DNMTs ability to catalyse the transfer of a methyl group to nearby cytosine residues.^79^ Oxidative damage commonly occurs at guanine residues, forming 8‐hydroxy‐2' ‐deoxyguanosine (8‐OHdG) and O6‐methylguanine but has also been shown to affect methylated cytosine residues. This forms the oxidative by‐product 5hmC. Since 5hmC is recognized poorly by DNMTs, DNAm may be lost at 5hmC sites during DNA replication. The putative impact of oxidative attack within CpG sites is depicted in Figure 3. DNA methylation is known to mediate chromatin organization via binding of methyl binding proteins (MBPs) that recruit histone modifying enzymes. Consequently, these interactions can result in chromatin condensation and transcriptional inactivation. Incorporation of 8‐OHdG and 5hmC in the MBP recognition sequence significantly inhibits the binding of MBP, meaning loss of DNAm may dysregulate multiple layers of epigenetic control.^80^ Since ROS are generated abundantly in response to UVR exposure, it is likely that at least part of the global loss of DNAm and dysregulation of chromatin assemblies, which is observed in photodamaged skin, may in part be driven by ROS attack on DNA.

### 3.2 Histone modifications directly affect genes associated with the pathogenesis of photoageing

Exposure to UVR significantly alters the activities of histone acetylation modulators. Global chromatin closure results from histone changes to prevent DNA replication and transcription initiation from damaged DNA, while local changes in acetylation could also dictate an open chromatin conformation for the DNA repair complex to access the damaged site. Indeed, Western blot and chromatin immunoprecipitation analysis of H3K9ac and H3K56ac has shown that these marks undergo rapid and reversible deacetylation in response to DNA damage in vitro, including following UVR exposure.^81^, ^82^ Similarly, in primary keratinocytes exposed to an acute solar‐simulated UVR dose, a global loss of H3K27ac was observed, including at promoter regions, alongside a decrease in the expression of HATs and HDACs, including Circadian Locomotor Output Cycles Kaput, sirtuin 1 (SIRT1) and HDAC4, 7 and 9.^11^ While many histone changes were found to be readily reversed 2 h after UVR exposure, H4K16ac was the only modification consistently reduced and transmitted through multiple rounds of cellular division.^82^ H4K16ac has been demonstrated to play a crucial role in higher‐order chromatin structure, and there is evidence of its hypoacetylation with increasing age in several tissues.^83^ As a whole, these results suggest that a global loss of H3 acetylation occurs following UVR exposure.

In contrast to the acute UVR response in keratinocytes, the acute UVR response in dermal fibroblasts showed an increase in H3 acetylation.^84^, ^85^ The role of P300 (a HAT) in the acetylation of H3 was confirmed in the fibroblast data, along with a role for p53 and gamma‐H2A histone family member X. In addition, within UVR‐exposed dermal fibroblasts, increasing levels of H3K4me3, a transcriptional activating mark, and a decrease in H3K9me2, a transcriptional silencing mark, were demonstrated on the promoters of *MMP1* and *MMP3*.^84^ Induction of MMPs is a well‐documented consequence of UVR, causing degradation of ECM components, including collagens, elastic fibres and fibronectin.

Corresponding to this, in the chronically sun‐exposed epidermis, significant increase in H3 acetylation was observed across the genome compared to that of photoprotected skin, which corresponded with an upregulation of P300 and a downregulation of SIRT1 and HDAC1.^86^ This acetylation correlated with the upregulation of several genes related to photoageing, including *MMP1*. This potentially provides an epigenetic mechanism by which transcriptional activation of genes strongly associated with photoageing are induced in response to UVR exposure through histone modifications. H3 acetylation changes were located to 308 gene promoters in the sun‐exposed epidermis, but not in photoprotected skin,^86^ highlighting how histone modifications may act as a conduit between UVR exposure, and resultant gene expression changes in response to UVR exposure and photoageing. A more recent study also observed an increase in global H3 acetylation levels with a decrease in HDAC activity following two doses of acute UVR exposure. These results are in direct opposition to the keratinocyte studies discussed, highlighting the importance of in vivo studies to allow the identification of trends that may not be recapitulated within in vitro models. In addition, the study found that an increase in H3 acetylation was also present in intrinsically‐aged skin compared to young skin.^87^ However, when comparing photoprotected skin to chronically photoexposed skin within elderly subjects, no significant differences in H3 acetylation levels were observed.^87^ These results are in opposition to the significant increase in H3 acetylation levels observed between photoexposed and photoprotected skin by Ding and colleagues,^86^ perhaps due to the fact that the subjects used in this study^87^ were comparably older (mean age of 76 compared to a mean age of 50), meaning that there was less divergence between the photoexposed and photoprotected body sites due to the changes in H3 acetylation that have already occurred with intrinsic ageing.

This section demonstrates the layers of epigenetic modulation that occur as a result of exposure to UVR. To summarise the results, it appears that within the skin, global H3 acetylation increases whilst HDAC activity decreases in response to acute UVR exposure, as well as chronic photoexposure, and intrinsic ageing; additionally, global hypomethylation of heterochromatic regions occurs within the epidermis following chronic photoexposure, in opposition to the slight trend in hypermethylation observed with intrinsic ageing (Table 1). However, the mechanism through which the changes are induced by UVR, such as through a direct response to UVR‐induced DNA damage, remains unknown, along with whether such changes are transient or only partly reversed following recovery from UVR exposure. Determining which of the acute changes persist will help to uncover the biological mechanisms at play that result in photoageing, and potentially aid in the design of interventions that can improve the associated decline in skin health and function.

## 4 EPIGENETIC MODIFICATIONS IN CANCER FORMATION, PROGRESSION, AND METASTASIS

Despite the known dangers of UVR exposure, the popularity of holidays to sunny destinations has led to a surge in skin cancer incidences. For example, between 1979 and 2015, the age‐standardized incidence rate of malignant melanoma rose from 5.75 to 30.44 per 100,000 people and is projected to increase a further seven percent by 2035.^88^ Understanding how the epigenome may change in response to skin cancer development and progression is essential, given the potential of epigenetic modifications to be used as disease biomarkers, as well as more amenable targets for therapeutic intervention compared to disease‐associated genetic variants.

It has long been regarded that DNA photodamage, predominantly in the form of cyclobutane pyrimidine dimers (CPDs) and, less frequently, 6‐4 photoproducts, plays an essential role in skin cancer induction. Ultraviolet radiation can directly damage the DNA, resulting in lesions which, if left either unrepaired or repaired incorrectly, become mutations, most commonly in the form of cytosine to thymine base substitutions.^89^, ^90^, ^91^, ^92^ Interestingly, methylated cytosines within dipyrimidine sequences are known to significantly enhance the formation of CPDs following UVB irradiation in particular,^93^, ^94^, ^95^ with the magnitude at which this occurs depending on the sequence flanking the CPD site.^96^ It is believed that this enrichment occurs due to differences in the absorbance spectra of cytosine and 5mC,^97^ the slow repair of CPDs involving 5mC^98^ and the enhanced deamination of 5mC involved in a CPD. As a result, mutational hotspots associated with cancer, such as within the tumour suppressor gene (TSG) *TP53*, occur preferentially at these trinucleotide sequences that incorporate a CpG site.^94^, ^95^ The effect of 5mC on the formation of 6‐4 photoproducts is unclear. Whilst one study found an increase in these lesions at methylated cytosines,^99^ another found a decrease, most likely due to the change in conformation of DNA with a methylated cytosine present, which favours CPD over 6‐4 photoproduct formation.^100^ Nevertheless, altogether this suggests that methylated cytosines are an important component in UVR‐induced mutagenesis and the subsequent development of skin malignancies.

### 4.1 Cutaneous melanoma

DNA methylation is possibly the most studied epigenetic mechanism involved in carcinogenesis. In many cancer types, including melanoma, hypermethylation of CGIs associated with numerous TSGs is an early event, often termed as the ‘CpG island methylator phenotype’.^101^ Hoon et al.^102^ identified four commonly hypermethylated genes in metastatic melanoma, of which at least one was found to be hypermethylated in 97% of tumours tested. These were *RASSF1A*, *MGMT*, *DAPK* and *RAR‐β2*, the latter of which was also found to be hypermethylated in an equal percentage of primary melanoma tumours. Aberrant DNAm in primary melanomas was also observed in a more recent study at several TSGs that are commonly silenced in cancer, most frequently at *RARB* (31% of patients) and *PTEN* (24%), with concomitant methylation often occurring between the two (15%).^103^ Methylation at these genes resulted in an increase in mitotic rate, growth rate of tumours, Breslow thickness, advancement in tumour stage, and a decrease in overall survival. This may, in part, be explained by the fact that the expression of the de novo methyltransferases DNMT3a and DNMT3b, which are essential in the methylation of tumour‐related genes, are increased in melanoma and correlate with the stage of disease and decreased overall survival.^104^, ^105^

Another study identified the *CDKN2A* gene, which encodes for two cyclin‐dependent kinase inhibitors, p15^INK4b^ and p16^INK4a^, and a regulator of the p53 pathway and p14^ARF^, as hypermethylated in a significant number of melanoma cases.^106^ This hypermethylation was associated with an increase in cell proliferation and reduced survival. In addition, Fujiwara et al.^107^ observed hypermethylation at several genes in melanoma samples, particularly at specific CpG sites within *NMP2*, a core histone chaperone associated with chromatin remodelling, demonstrating how aberrant DNAm can alter gene expression directly or indirectly through influencing other epigenetic modifications. Furthermore, an increase in hypermethylation was observed at gene promoters in metastatic melanoma compared to non‐metastatic melanoma and pre‐malignant melanocytes,^108^ particularly at developmental genes, similar to that observed by Tilburg and colleagues^12^ in response to UVR exposure, potentially revealing a link between cumulative UVR exposure and melanoma progression. It would be interesting to determine whether the datasets from these studies do indeed show any similarities in differentially methylated CpG sites. As a whole, it was found that malignant melanomas that were highly methylated in gene promoters resulted in a significantly worse prognosis and increased tumour thickness compared to tumours with less frequent hypermethylation at promoter CpG islands.^109^

In addition to site‐specific hypermethylation, methylation levels are depleted globally in the genome in many cancers, mainly in heterochromatic regions such as centromere repeats and other repetitive sequences; this is thought to contribute towards chromosome instability, activation of endogenous retroviral elements, oncogene activation and immune evasion.^110^, ^111^, ^112^, ^113^ Hypomethylation of long interspersed nuclear element (LINE)‐1 can be used as a surrogate for assaying global methylation levels and robustly correlates with poor outcome in some cancers, including oesophageal, gastric and liver cancers.^114^, ^115^, ^116^ In melanoma cell lines, LINE‐1 is hypomethylated compared to normal epidermal melanocytes.^117^ Furthermore, the level of hypomethylation of LINE‐1 in the different melanoma cell lines was positively correlated with the number of hypermethylated genes identified.

In addition to global 5mC depletion, 5hmC levels and the expression of enzymes responsible for converting 5mC to 5hmC (TET1‐3) have also been found to be significantly reduced in melanomas compared with normal melanocytes and benign melanocytic nevi, corresponding to increased dysplasia and poor clinical outcome.^118^, ^119^ Further to this, a subset of genes with strong associations to numerous cancer pathways were identified; these genes were hypermethylated, whilst simultaneously showing a decrease in 5hmC levels in melanomas compared to benign nevi.^119^ With this in mind, it is possible that measuring both the global loss of both 5mC and 5hmC levels, as well as the 5mC and 5hmC status of specific genes, could assist in screening and prognosis prediction for melanoma patients. In addition, this research study could aid the identification of potential therapeutic targets, such as TET enzymes,^119^ to impede melanoma progression and improve patient outcomes.

The loss of methylation in heterochromatic regions is similar to that observed in aged skin, indicating that some of the epigenetic changes that are evident in cancer cells are already underway in ageing. For example, senescent cells display loss of methylation in heterochromatic regions, including repetitive elements, and display increased retrotransposon activity.^120^ Such cells are thought to be pre‐cancerous and their similar epigenetic profile to cancer cells highlight that their removal could help reduce the risk of skin forming cancerous growths. The testing of senolytics in humans is underway and has been proven to reduce the number of p16‐ and p21‐positive cells (markers of cell senescence) in the skin.^121^ Further research could help establish whether the epigenetic modifications evident in senescent cells offers a route to target these cells without harming surrounding non‐senescent cells. Follow‐up studies on whether current senolytics reduce the incidence of skin cancer are required to determine their ability to address morbidity in elderly populations.

Histone modifications have also been consistently observed in melanoma cells and have been correlated to cellular and clinicopathological features of disease. One of the most commonly studied histone modifications in melanoma is acetylation, in which aberrant hypoacetylation is observed.^122^ Inhibition of HDACs via trichostatin A in a melanoma cell line was shown to increase apoptosis through upregulation of the cyclin‐dependent kinase inhibitor, p21, in a dose‐dependent manner.^122^ Another HDAC inhibitor, suberic bishydroxamate, was also found to induce apoptosis in melanoma cell lines compared with normal melanocytes and fibroblasts, via the downregulation of anti‐apoptotic proteins (e.g. Mcl‐1 and Bcl‐XL) and the upregulation of pro‐apoptotic proteins (e.g. Bim, Bax, and Bak).^123^ Another study identified a downregulation of *PIB5PA* in melanoma via hypoacetylation, mediated by interaction of HDACs with the transcription factor sp1, which binds the *PIB5PA* gene promoter.^124^ Overexpression of PIB5PA in vitro blocked PI3K/Akt signalling, thereby inhibiting proliferation of melanoma cells. As a whole, this suggests that hypoacetylation (and therefore, downregulation) of TSGs involved in apoptotic pathways and proliferation inhibitors may contribute to melanoma pathogenesis, resulting in an increase in proliferation and growth of melanocytes.

In addition to histone hypoacetylation, histone methylation is believed to play a role in melanoma progression. In a metastatic murine cell line, H3K36me2 levels were reduced compared to cell lines modelling the earlier stages of melanoma, whilst H3K36me3 levels were increased, similar to that seen in metastatic colon cancer; this was in addition to several other histone methylation changes, including a reduction in H3K79me2, H3K23me1 and H3K18me1, and an increase in H3K9me2.^125^ Enhancer of zeste homologue 2 (EZH2) is the enzymatic subunit of the polycomb repressive complex (PRC2), responsible for catalysing the transcriptionally repressive methylation of H3K27. Upregulation of EZH2 is robustly associated with highly proliferative and aggressive melanoma subtypes, and has been shown to increase incrementally from benign nevi to melanoma and invasive subtypes as shown by immunohistochemical analysis.^126^, ^127^ The INK4b‐ARF‐INK4a locus, which encodes the TSGs p15 and p16, is a known target of EZH2, as well as other epigenetic regulators, including histone demethylases and DNMTs,^128^ demonstrating that multiple levels of the epigenome (e.g. DNAm of the promoter and deposition of repressive histone marks) can act in concert to silence target genes.

The histone methyltransferase, SETDB1, is also upregulated in melanoma but not in benign nevi and normal melanocytes.^129^ SET domain bifurcated 1 catalyses the trimethylation of histone H3K9, a repressive histone mark that leads to downregulation of target genes. In a zebrafish melanoma model, an increase in SETDB1 expression significantly accelerated melanoma formation and transcriptionally deregulated a number of genes, including HOX genes,^129^ which are also associated with melanoma prognosis through DNAm changes. Amplification of the *SETDB1* locus drives its expression upregulation, potentially implicating LINE‐1 retrotransposon activity as a driver of such changes due to their association with genomic amplification.^130^ The inhibition of SETDB1 was found to significantly reduce cell viability in melanoma, therefore suggesting that this enzyme has the potential to become as yet another novel therapeutic target.^131^

To summarise, melanoma development and progression is associated with a number of epigenetic events, with an overall global loss of 5mC and 5hmC alongside site‐specific hypermethylation of TSGs, and a range of aberrant histone modifications (Table 1). Each of these have the potential to be used as biomarkers to aid the diagnosis of melanoma cases and predict the clinical outcome of the patient. Further insights into the mechanisms in which these epigenetic marks interact with each other and the genome will enable the identification of more therapeutic targets in addition to those already identified.

### 4.2 Non‐melanoma skin cancers

Squamous cell carcinomas and BCCs represent the majority of skin cancer cases and are implicated as the most common type of cancer in developed countries. However, as these skin cancers are less aggressive than cutaneous melanoma, and are often resolved by surgical excision, investigations into their aetiology are more limited.

#### 4.2.1 Cutaneous squamous cell carcinoma

A study examining methylation levels of repetitive sequences in lesional and perilesional cutaneous SCC tumour samples found lower methylation levels, including in LINE‐1 loci, suggestive of a global trend towards hypomethylation.^132^ Though this was not significant, studies in mice also identified progressive global hypomethylation in response to long‐term UVR exposure^133^ and topical application of various mutagens and tumour promoting agents.^134^ Global loss of DNAm occurred during the transition from non‐tumourigenic to benign papilloma cells and between transformed cells undergoing an epithelial to spindle transition.^134^ This is in contrast to another mouse study, which suggested that SCC tumours may be characterised by global hypermethylation^135^; however this conclusion was derived from the analysis of <0.4% of all mouse CpGs and is therefore unlikely to be representative of the entire genome. A more recent study investigating human subjects found that whilst global loss of DNAm occurred between actinic keratosis and low‐risk SCC, global hypermethylation then occurs in the transition from low‐risk to high‐risk SCCs.^136^ For example, of the 637 differentially methylated CpGs identified between pre‐malignant and malignant SCCs, 602 CpGs were hypermethylated and just 35 were hypomethylated,^136^ suggesting an association between increasing methylation with a more high‐risk tumour evolution.

Candidate gene approaches have identified promoter hypermethylation of TSGs in SCC, similar to that observed in melanoma. Hypermethylation of *DAPK1* increases progressively between the sun‐protected and sun‐exposed normal skin and the perilesional and lesional skin.^132^ Other candidate genes, such as *CDH13*, are exclusively hypermethylated in SCC compared to photoexposed and photoprotected skin samples.^132^ In addition, hypermethylation of *FRZB*, which encodes a member of the Wnt signalling pathway, correlated with metastasis of SCC.^137^ Furthermore, the *FILIP1L* TSG was hypermethylated and downregulated in both murine and human SCC^135^; this gene is also involved in Wnt signalling,^138^ although its exact role in the skin and SCC is unknown. Hypermethylation events have been associated with an increase in the expression of DNMT1 and DNMT3b in mouse skin carcinogenesis.^134^ In this study, hypermethylation of *MGMT*, *Snail* and *MLT1* promoters occurred during the transition from normal to immortalised non‐tumourigenic keratinocytes. *E‐cadherin* hypermethylation occurred in the latter stages of tumour progression, coinciding with an epithelial to spindle transition. Hypermethylation of these genes was further associated with a loss of H4Ac and H3K4me2, demonstrating perturbations at multiple levels of the epigenetic landscape are relevant to carcinogenesis.^134^ The mechanisms targeting hypermethylation to these tumour suppressor promoters, whilst other loci undergo hypomethylation, however, are unclear and need dissecting.

With regard to histone modifications, differential H3K27ac has been associated with SCC and was found to be significantly enriched in regions of the genome involved in the immune response, suggesting that epigenetic changes in SCC contributes to dysregulated immune function.^139^ In support of this, an increase in the histone methyltransferase, EZH2, in metastatic SCC compared to non‐metastatic tumours was associated with an impairment in innate immunity that enabled the escape of tumour cells from the immunosurveillance system to result in metastases.^140^ In addition, cancerous stem cells within SCC tumours, which improve the tumour's ability to invade tissues and migrate, have been shown to require increased EZH2 and its associated mark, H3K27me3, for optimal survival.^141^ Alongside EZH2, numerous other regulators of histone modifications have been linked to SCC, including the overexpression of the histone demethylase, *KDM1A*,^142^ and high mutation frequencies of the histone methyltransferase, *KMT2D*,^143^ the histone demethylase, *KDM6A* ^144^ and acetyltransferases *p300* and *CBP*,^145^ amongst several others. Inhibition or knockdown of EZH2 and KDM1A may therefore suppress tumour formation, as well as therapies that target dysregulated pathways that are related to frequently mutated epigenetic modifiers.

#### 4.2.2 Basal cell carcinoma

Given the rarity in which BCCs become metastatic, the epigenetic biology behind this malignancy has not been extensively studied, and a limited number of TSGs have been investigated by candidate‐gene approaches. Fragile histidine triad *(FHIT)* is a TSG involved in apoptotic pathways and is commonly inactivated in a number of human cancers; its inactivation may be due to hypermethylation of its promoter.^146^ Hypermethylation within the promoter region of *PTCH* has also been detected in 31% of BCCs tested,^147^ whilst genetic mutations in *PTCH* have been found in 67% of sporadic cases,^148^ suggesting that genetic mutations might be more important drivers of PTCH dysregulation than epigenetic changes.

Similar to SCC, BCC also showed aberrant H3K27ac levels compared to non‐tumourigenic skin.^139^ In contrast to SCC, the differential H3K27ac was significantly enriched in genes responsible for several processes, including epidermal and hair follicle proliferation and maintenance, suggesting that abnormal epidermal homoeostasis contributes more to the development of BCC than the immune dysfunction observed in SCC. In addition, the upregulation of EZH2 and H3K27me3 was observed in BCCs compared to non‐malignant keratinocytes: interestingly, an upregulation of EZH2 was identified in aggressive BCCs compared to milder BCCs, whilst increased H3K27me3 was positively correlated with milder BCCs, despite the fact that EZH2 is responsible for catalysing H3K27me3 deposition.^149^ This observation appears paradoxical, but this pattern has also been observed in other cancer types.^150^

This demonstrates the wide range of epigenetic dysregulation that occurs during the development of skin malignancies. Similar epigenetic mechanisms can be observed across all skin cancer types, such as the hypermethylation of TSGs, with each subtype also exhibiting distinct molecular signatures (Table 1). By understanding these mechanisms for each type of cutaneous malignancy, epigenetic biomarkers may be selected and utilised in distinguishing malignant cells from benign, predicting prognosis and determining the likelihood of therapeutic resistance. The potential therapeutic benefit of agents which target these alterations is being investigated, with several ongoing clinical trials looking into skin cancer treatments that can target the epigenetic dysregulation observed.^151^ The challenge will be ensuring that these therapies are efficacious whilst minimising off‐target effects, as well as determining which patients will benefit from these treatments over the standard practice targeted therapies and immunotherapies.

## 5 CONCLUSIONS

Exposure of the skin to UVR contributes to photoageing and is a risk factor for skin cancers via the induction of oxidative stress, DNA damage and inflammatory responses. This combination has been demonstrated to markedly alter epigenetic machinery at all layers of the epigenetic landscape and is likely involved in triggering epigenetic changes in skin cells. An important question is how pervasive are the epigenetic changes that accompany acute exposure to UVR? Furthermore, are these changes causative in the chronic consequences of UVR? From a public health perspective, the research discussed in this review is vital for dissecting the biological mechanisms behind photoageing and directing future research studies investigating the prevention or rejuvenation of diseases and phenotypes associated with aged skin. Epigenetic changes have proven to be useful biomarkers for exposure and disease, and epigenetic regulators are currently being investigated for their potential use as therapeutic targets for skin cancer treatments. However, many challenges still remain in the measurement and interpretation of epigenetic changes and in achieving actionable interventions.

## CONFLICT OF INTEREST STATEMENT

DAG is an employee of Unilever R&D UK. BMB and AS were/are supported by BBSRC iCASE studentships awarded to Unilever R&D UK.

## AUTHOR CONTRIBUTIONS

**Bethany M. Barnes**: Conceptualization (equal); writing – original draft (equal). **Anya Shyne**: Conceptualization (equal); writing – original draft (equal). **David A. Gunn**: Conceptualization (equal); supervision (equal); writing – review & editing (equal). **Christopher E. M. Griffiths**: Conceptualization (supporting); supervision (equal). **Rachel E. B. Watson**: Conceptualization (equal); supervision (equal); writing – review & editing (equal).

## ETHICS STATEMENT

Not applicable.

## PATIENT CONSENT

Not applicable.

## Data Availability

Data sharing not applicable to this article as no datasets were generated or analysed during the current study.
